# Never Too Late: A Case Report on Transcatheter Aortic Valve Implantation in a 97-Year-Old Patient

**DOI:** 10.3390/geriatrics2030025

**Published:** 2017-07-17

**Authors:** Mihaela Zegrean

**Affiliations:** Harper University Hospital, Detroit, MI 48201, USA; lorezeg@gmail.com; Tel.: +1-313-719-8421

**Keywords:** aortic valve stenosis, transcatheter aortic valve implantation, nonagenarians

## Abstract

Aortic valve stenosis is a well-recognized valvular problem in the aging population. Transcatheter aortic valve implantation (TAVI) is becoming an increasingly popular treatment alternative to surgical aortic valve replacement for frail elderly individuals with symptomatic severe aortic valve stenosis. There are multiple research reports documenting the effectiveness of TAVI in octogenarians; however, few authors discuss the success of this procedure in nonagenarians. This case report depicts the successful transfemoral implantation of a prosthetic aortic valve in a 97-year-old man. Moreover, the current literature on TAVI outcomes in nonagenarians is reviewed.

## 1. Introduction

Stenosis of the aortic valve is a common type of valvular heart disease in the elderly due to degenerative changes of the valve [1]. The prevalence of aortic stenosis in North Americans older than 75 years is 2.7 million [2]. Of these, 540,000 individuals will develop severe symptomatic aortic stenosis [3], which manifests as chest pain, syncope with exertion, and congestive heart failure [4]. This drastically reduces quality of life and increases mortality risk. Although surgical aortic valve replacement (SAVR) has been the standard treatment for severe symptomatic aortic stenosis, this approach is controversial in the elderly population, as peri-operative death rates approach 10% in those beyond 90 years of age [3]. This is concerning, as the United States’ population is comprised of 1.9 million nonagenarians who live with various disabilities, including aortic valve stenosis [5]. Furthermore, the nonagenarian population is expected to quadruple by the year 2050 [5].

Transcatheter aortic valve implantation (TAVI) has been a feasible alternative to SAVR in those with symptomatic severe aortic stenosis and high mortality risk [3]. Alain Cribier pioneered this treatment approach in France in 2002 [6]. Based on two multicenter North-American trials—namely, PARTNER 1 [7] and PARTNER 2 [8]—which showed comparable results in those undergoing TAVI versus SAVR with regard to symptomatic improvement and all-cause mortality, the procedure was later approved by the United States’ Food and Drug Administration in 2011 [6]. According to the 2017 focused update guideline of the American Heart Association and the American College of Cardiology, TAVI is a class I recommendation for severe symptomatic aortic stenosis in patients who are at high risk for surgical valve replacement [9]. While TAVI has frequently been performed in octogenarians, there is limited literature on its effectiveness in nonagenarians. The author of this article will report on the successful outcome of TAVI in a 97-year-old North-American man. Furthermore, a review of the literature on the therapeutic effectiveness of this procedure in the nonagenarian patient population will be included.

## 2. Case Report

In June 2016, a 97-year-old man presented to the cardiology clinic with a feeling of impending doom and symptoms of heart failure New York Heart Association class III (dyspnea with minimal exertion, peripheral edema, and fatigue) after recently being treated in the emergency department for similar symptoms with intravenous diuretics. The patient had a long-standing history of asymptomatic severe aortic stenosis and had been highly functional until that day. Three years prior, he was denied SAVR due to being considered a high surgical risk. A 2D echocardiogram revealed a trileaflet aortic valve with a valve area of 0.5 cm^2^ (normal is 3–4 cm^2^) and a mean transvalvular gradient of 48 mmHg (normal is <5 mm Hg), which indicated severe aortic valve stenosis. Additional co-morbidities consisted of moderate tricuspid regurgitation, hypertension, chronic obstructive pulmonary disease (COPD), chronic renal disease stage III, gastrointestinal hemorrhage in 2013, and adenocarcinoma of the prostate that was treated in 1991 with radiation and adjuvant hormone therapy. On assessment, his blood pressure was 143/70 mm Hg, heart rate was 50 beats per minute, respiration rate was 14 breaths per minute, and he was afebrile. Auscultation of the heart revealed the class murmur of aortic valve stenosis, which was a loud ejection murmur over the aortic area, radiating to the carotid arteries. He had bilateral lower extremity edema, +2, and non-pitting. 

### 2.1. Preoperative Evaluation for TAVI

The patient was admitted to the hospital emergently. His pre-operative risk assessment for 30-day mortality—the Society of Thoracic Surgeons (STS) score—was elevated at 14.4% [10], and he was thus evaluated for TAVI. Multiple tests were performed to assess the feasibility of the procedure. CT angiograms of the thorax, abdomen, and pelvis were implemented to investigate for abnormalities of the vasculature that would prohibit a transfemoral approach for TAVI. Considering that stroke is a common complication of this procedure [6], a carotid ultrasound was performed to evaluate for carotid atherosclerosis. Two cardiothoracic surgeons examined the patient and declared that he would be at high mortality risk to have SAVR, and thus they recommended TAVI. Cardiac catheterization was performed to evaluate for coronary artery disease and to obtain hemodynamic measurements.

### 2.2. Performance of TAVI

**Figure 1:**
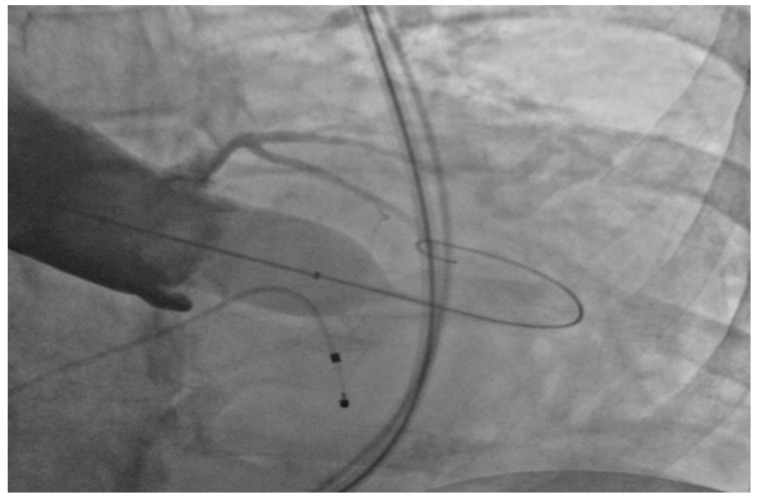
Balloon inflation across the aortic valve.

**Figure 2:**
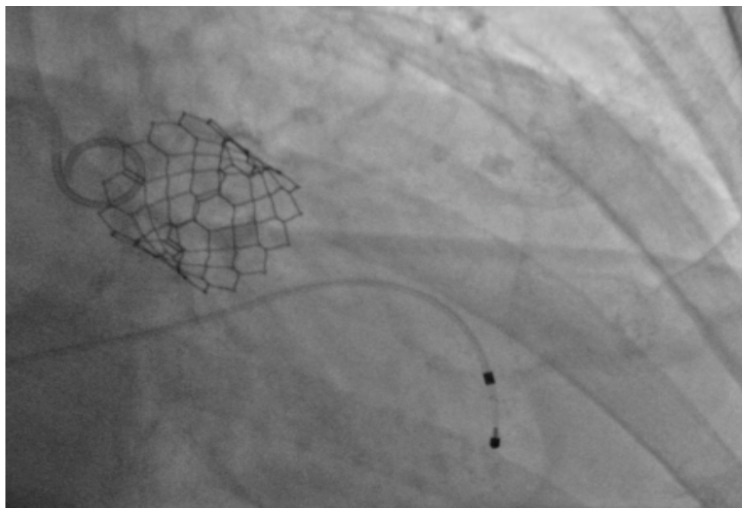
Expanded SAPIEN S3 valve within the native aortic valve.

Under general anesthesia, the right and left femoral arteries were each accessed with 6-french sheaths. A temporary pacemaker was placed in the right ventricle through an 8-french sheath in the right femoral vein. Balloon valvuloplasty was performed by advancing a balloon via the right femoral artery sheath, and during rapid ventricular pacing at 160 beats per minute, inflating it across the aortic valve to clear the stenosis and to deploy the 26-mm SAPIEN S3 bioprosthetic aortic valve (Figure 1), which expanded within the native aortic valve (Figure 2). The purpose of rapid ventricular pacing during TAVI is to reduce cardiac output, which facilitates balloon inflation across the valve and placement of the bioprosthetic aortic valve. The mean valvular gradient after TAVI decreased to 1.9 mm Hg (normal is <5 mm Hg). There were no intraoperative complications. The patient was extubated and transferred to the coronary care unit with the temporary transvenous pacemaker, which was removed two days later. 

### 2.3. Postoperative Course

A 2D echocardiogram performed on the first postoperative day showed that the prosthetic aortic valve was well seated without any regurgitation. A 12-lead electrocardiogram revealed new onset paroxysmal atrial fibrillation with slow ventricular response (his heart rate was in the range of 50 beats per minute). Anticoagulation treatment for the prevention of thromboembolic events was initiated with Apixaban 2.5 mg BID. The lower dose of Apixaban was selected because he was older than 80 years and his serum Creatinine level was above 1.5 mg/dL [11]. In addition, Clopidogrel 75 mg daily was started to prevent stenosis of the bioprosthetic valve. The patient was discharged home three days post procedure.

### 2.4. Follow-up Visits

One month later, during a follow-up appointment with the primary care provider, the patient was found to be severely bradycardic and became unresponsive for a few minutes. He regained consciousness without any resuscitative efforts and was taken emergently to the hospital. An inpatient limited 2D echocardiogram showed normal systolic function with ejection fraction of 55–60%. Unfortunately, nothing was reported on the function of the bioprosthetic aortic valve. The patient remained asymptomatic during the hospitalization and was discharged home the next day. A review of patient’s home medications revealed that he was taking the negative chronotropic medication metoprolol succinate, which may have precipitated his syncopal episode. He was instructed to stop this medication. 

During the six-month follow-up visit, the patient reported continued symptomatic improvement. He had mild peripheral edema. Dyspnea occurred with more significant exertion; thus, NYHA functional class II. He remained off metoprolol as instructed, and despite being bradycardic with a heart rate of 55 beats per minute, he did not experience any further episodes of dizziness. A limited 2D echocardiogram revealed that the bioprosthetic valve was well seated without any paravalvular leak. The ejection fraction was 65% and he had mild diastolic dysfunction. The patient was told to stop clopidogrel (as he had completed the standard six-month treatment), and to continue antiplatelet therapy with Aspirin 81 mg daily indefinitely. 

## 3. Literature Review

There were nine articles in the literature that addressed TAVI in nonagenarians, which included four case reports and thirteen research studies. Most participants in the studies were women. Patient selection for TAVI was generally based on operative mortality risk, which was calculated using the Society of Thoracic Surgeons (STS) score or the European System for Cardiac Operative Risk Evaluation (EuroSCORE); however, a few authors mentioned the importance of also using determinants of quality of life such as physical functioning status, social support, and cognitive status [12,13,14,15]. The Duke Activity Status Index was mentioned in two TAVI studies for the evaluation of physical functioning [12,13]. The Kansas City Cardiomyopathy Questionnaire (KCCQ-12) was another instrument used to evaluate the impact of cardiac symptoms on quality of life [15]. 

The author of the earliest case study documented the performance of TAVI via the transapical approach in a 96-year old woman with positive outcomes at one-month follow-up [11]. Jabs et al.’s case report illustrated the success of TAVI in a 99-year old patient with multiple comorbidities, whose valve function and physical functioning were excellent at five-year follow-up [12]. Similar findings were noted in Kneitz et al.’s case study of a 95-year-old woman [16]. The oldest patient known from the literature to have had TAVI was a 102-year-old woman [13]. Approximately four years later, transthoracic echocardiography revealed good functioning of her bioprosthetic aortic valve with mild paravalvular aortic regurgitation. In addition, she was able to perform activities of daily living without any assistance [13]. 

### 3.1. Complications after TAVI

The most common procedural complications in nonagenarians were vascular in nature and were significantly more likely to require surgical intervention than in the participants younger than 90 years [14,15,16,17,18,19,20,21,22,23,24,25,26,27,28]. Gurvitch et al. reported that post-procedural bleeding was most frequently due to Coumadin therapy for atrial fibrillation [20]. Buchanan et al. discussed the occurrence of myocardial infarction in the perioperative phase due to dissection of the left main coronary artery after it was balloon dilated [23]. Other common in-hospital complications of TAVI included high-grade atrioventricular block requiring pacemaker implantation, atrial fibrillation, and stroke, respectively [12,13,14,15,16,17,18,23,24,25,26,27,28]. Nonagenarians were more likely to require transfer to the intensive care unit and to be discharged to an extended care facility as compared to their younger counterparts [15]. They also had higher readmission rates at 30 days post TAVI due to heart failure [15].

### 3.2. Mortality after Hospital Discharge

There were great variations in the death rates post TAVI, which were likely impacted by the sample sizes of the research articles. The 30-day all-cause mortality rates ranged from 0% to 27% [15,17,18,19,21,23,24,25,26,27], while the one-year mortality rates were between 10–32% [15,17,18,19,21,23]. However, survival at 5-year follow-up was 30.4% [25]. Most comparison studies revealed that mortality rates at one year were similar in nonagenarians versus the younger cohorts in [19,20,21,24]. However, Scholtz et al. found that nonagenarians had significantly higher death rates at 30 days and one-year follow-up [24]. Despite enrolling a healthier nonagenarian population, Escarcega et al. reported that death rates at 30 days were higher than in the octogenarian cohort [21]. Moreover, death rates at six months post TAVI were noted to be double in Yamamoto et al.’s nonagenarian patient population as compared to their patient group younger than 90 years [18]. This was attributed to extensive cardiovascular comorbidities such as moderate to severe mitral regurgitation and NYHA functional class IV. Most non-survivors had previous aortic valvuloplasty and congestive heart failure within the preceding 12 months [18]. Causes of death included hemorrhage, pneumonia, heart failure, stroke, and sudden death [18]. Similar findings were reported by Gurvitch et al., who found that deaths post TAVI were primarily due to respiratory problems, such as respiratory failure, pneumonia, and chronic obstructive pulmonary disease [20]. Escarcega et al. reported that variables associated with 30-day mortality in nonagenarians were hemorrhage, stroke, and post-TAVI implantation of pacemaker [21]. The factors correlated with one-year mortality in this patient population were the same as those at 30-day with the addition of moderate aortic valve regurgitation [21]. The transfemoral versus the transapical approach was a predictor of lower 30-day mortality [21]. Furthermore, male gender and renal failure were found to be mortality predictors [25].

### 3.3. TAVI in Centenarians

Although the scope of this literature review is to describe TAVI in nonagenarians, it is worth illustrating the success of this procedure by mentioning the outcomes in a sample of 24 centenarians from Arsalan et al.’s research study [15]. Post-procedural complications in these patients were primarily vascular in nature and more frequent as compared to the younger cohort. There were no deaths at 30 days, and the one-year mortality was 6.7% [15]. 

## 4. Discussion

It is estimated that there are 100,000 candidates for TAVI in North America [2]. Favorable results were reported in octogenarians undergoing TAVI [3]. The literature base on TAVI in nonagenarians is presently limited. Most of the articles described above depicted the effectiveness of TAVI in European subjects. However, the success rate of this procedure is generally unknown in North American nonagenarians. There are multiple approaches to TAVI (i.e. transfemoral, transapical, transaortic, subclavian, transcarotid), but the transfemoral route was most frequently implemented in the nonagenarian populations from the reviewed articles [29]. This was likely due to the less-invasive nature of the transfemoral approach. 

Comparison of TAVI versus SAVR outcomes in nonagenarians was very limited in the research literature. There were similar lengths-of-stay and mortality rates in-hospital and at one year follow-up [30,31,32]. Those who underwent SAVR were more likely to experience renal failure and to require blood transfusions [30]. Patients in both treatment groups had improved quality of life at one year post-procedure [31]. However, given the less-invasive nature of TAVI in comparison to SAVR, its popularity will likely continue to increase, which will prompt further research in this patient population. Suggestions for future research include studies with larger samples and long-term follow-up that compare the effectiveness of the various types of bioprosthetic aortic valve as well as the patients’ quality of life. Furthermore, standardization of geriatric assessment pre- and post TAVI to determine quality of life would be beneficial in determining the success of this procedure across health care centers in various geographic locations.

Although TAVI seems to be a promising alternative for those who are too frail for SAVR, the financial impact of this procedure on the health care system has been a rarely evaluated variable in the literature. A cost analysis of transfemoral TAVI based on the PARTNER trial cohort B revealed a procedural cost of $42,806 and a hospitalization cost of $78,542 [33]. These costs were higher than those associated with standard nonsurgical therapy [33], conventional SAVR [34], and the newer sutureless technique for SAVR [35]. The incremental cost effectiveness ratio for TAVI was $502,000 per year of life saved, which was deemed acceptable according to US healthcare spending thresholds [34]. However, there were no cost effectiveness analyses of this procedure in nonagenarians, despite its increasing frequency and high number of comorbidities in this patient population. Furthermore, when considering costs and therapeutic success, careful deliberation must be given to potential complications and anticipated quality of life post-procedure. The only complication experienced by the patient described in this case report was atrial fibrillation. His physical functioning after TAVI was excellent, and he had extensive social support.

## 5. Conclusions

As the life expectancy continues to rise, especially in developed nations, and more individuals survive into the tenth decade of life and beyond, there is a need for less invasive treatments that add quality to longevity. TAVI is a revolutionary approach to symptomatic severe aortic stenosis, which carries a grim prognosis for those who do not qualify for surgical valve replacement. The current case report of the 97-year-old man demonstrates that it is never too late to push the boundaries of medicine in the new millennium.
